# Utility of primary cells to examine NPC1 receptor expression in *Mops condylurus*, a potential Ebola virus reservoir

**DOI:** 10.1371/journal.pntd.0007952

**Published:** 2020-01-21

**Authors:** Marcel Bokelmann, Kathryn Edenborough, Nicole Hetzelt, Petra Kreher, Angelika Lander, Andreas Nitsche, Uwe Vogel, Heinz Feldmann, Emmanuel Couacy-Hymann, Andreas Kurth

**Affiliations:** 1 Centre for Biological Threats and Special Pathogens, Robert Koch Institute, Berlin, Germany; 2 Laboratory of Virology, Rocky Mountain Laboratories, National Institute of Allergy and Infectious Diseases, National Institutes of Health, Hamilton, MT, United States of America; 3 Laboratoire National d'Appui au Développement Agricole, Bingerville, Ivory Coast; Saudi Ministry of Health, SAUDI ARABIA

## Abstract

The significance of the integral membrane protein Niemann-Pick C1 (NPC1) in the ebolavirus entry process has been determined using various cell lines derived from humans, non-human primates and fruit bats. Fruit bats have long been purported as the potential reservoir host for ebolaviruses, however several studies provide evidence that *Mops condylurus*, an insectivorous microbat, is also an ebolavirus reservoir. NPC1 receptor expression in the context of ebolavirus replication in microbat cells remains unstudied.

In order to study Ebola virus (EBOV) cellular entry and replication in *M*. *condylurus*, we derived primary and immortalized cell cultures from 12 different organs. The NPC1 receptor expression was characterized by confocal microscopy and flow cytometry comparing the expression levels of *M*. *condylurus* primary and immortalized cells, HeLa cells, human embryonic kidney cells and cells from a European microbat species. EBOV replication kinetics was studied for four representative cell cultures using qRT-PCR. The aim was to elucidate the suitability of primary and immortalized cells from different tissues for studying NPC1 receptor expression levels and their potential influence on EBOV replication.

The NPC1 receptor expression level in *M*. *condylurus* primary cells differed depending on the organ they were derived from and was for most cell types significantly lower than in human cell lines. Immortalized cells showed for most cell types higher expression levels than their corresponding primary cells. Concluding from our infection experiments with EBOV we suggest a potential correlation between NPC1 receptor expression level and virus replication rate *in vitro*.

## Author summary

Although there have been Ebola virus (EBOV) outbreaks for more than 40 years, the animal natural reservoir that maintains this virus in nature has not been identified. Viruses and their respective reservoirs coevolve over millions of years, often without causing diseases in the reservoir itself. Upon entering a new host, infection can have devastating consequences, as in the case of EBOV. To gain entry into cells prior to replication, all ebolaviruses utilize the cellular receptor Niemann-Pick C1 (NPC1). In this study the authors focus their work on the Angolan free-tailed bat (*Mops condylurus*) as a potential reservoir for EBOV. Cells from various organs of this bat were isolated in culture and tested for the presence of NPC1. Most bat cell types had a lower amount of NPC1 compared to the tested human cells. These bat cells were also less efficiently infected by EBOV, indicating adaptation to EBOV. These results suggest low levels of virus replication in the respective tissues of *M*. *condylurus* and might be indicative of a virus-natural reservoir relationship.

## Introduction

*Ebolavirus* and *Marburgvirus* are genera within the family *Filoviridae* in the order of Mononegavirales [1]. Six species within the *Ebolavirus* genus have been discovered: *Zaire*, *Sudan*, *Taï Forest*, *Bundibugyo*, *Reston* and most recently *Bombali ebolavirus*. Four of these (*Zaire*, *Sudan*, *Taï Forest* and *Bundibugyo ebolavirus*) are known to cause severe hemorrhagic fever in humans with case fatality rates up to 90% [1–3]. Since 1976, 28 ebolavirus outbreaks have been documented in Africa and between 2014–2016 the largest outbreak caused by Ebola virus (EBOV) occurred, resulting in over 28,600 cases [4]. The second largest outbreak in history is currently ongoing in the Democratic Republic of the Congo (DRC) [5], until October 2019 resulting in over 3200 cases [6].

Outbreak investigations and several epidemiological studies provided evidence that bats are most likely the natural reservoir host for ebolaviruses [7, 8]. In rural parts of Africa interactions between humans and bats occur regularly [9, 10] and for several outbreaks there is anecdotal evidence of index patients contacting bats prior to infection [7, 11, 12]. Various species of wild-caught bats have been serologically tested for EBOV seroreactivity, which has been detected in 307 individual bats from at least 17 species in Africa and Asia [7, 13–21]. EBOV RNA has been detected in three fruit bat species (*Hypsignathus monstrosus*, *Epomops franqueti* and *Myonycteris torquata*) in 2005 [13], which has led to a sampling bias towards fruit bats, while insectivorous bats have received only sparse attention in ebolavirus research [22]. Recent discovery of a new ebolavirus, Bombali virus (BOMV), in two microbat species from Sierra Leone (*Mops condylurus* and *Chaerephon pumilus)* [23] indicate that further investigation into the role of microbats in ecology of ebolaviruses is required. Before the discovery of BOMV, there have been several studies providing evidence that the species *M*. *condylurus* is a potential reservoir of ebolaviruses [11, 15, 24].

A key component of the filovirus entry process is the protein Niemann-Pick C1 (NPC1). NPC1, an integral membrane protein found in late endosomes and lysosomes [25], is highly conserved within mammalia [25] and is ubiquitously expressed in human cells [25, 26]. Human tissues or cell lines without NPC1 expression are not described from healthy donors. NPC1 mediates the intracellular trafficking of cholesterol [27] and mutations in the NPC1 gene ensue in defective cholesterol export from lysosomes leading to Niemann-Pick C1 disease, a fatal neurodegenerative disorder [28–30]. Utilized in the viral entry process by the glycoproteins (GP) of Ebola virus [31–33], Marburg virus [34] and Měnglà virus (MLAV) [35], NPC1 is essential for membrane fusion and egress of virus particles from late endosomes into the cytosol. NPC1 knockout cells are refractory to infection with EBOV [25, 27]. In bat cells it was also shown that NPC1 is a genetic determinant of filovirus susceptibility as NPC1 polymorphisms found in specific bat species led to reduced interactions between filoviruses and NPC1, which might reflect host adaptation [36]. These interactions influence the cellular susceptibility of bats to infection, filovirus replication and virulence [36]. The NPC1 expression levels throughout human tissues and human cell lines differ significantly [37–40]. In particular, human cell lines show high NPC1 expression levels.

The examination of EBOV entry and infection processes in bat cells have predominantly been performed in immortalized cell lines [36, 41–49]. For the purpose of having an informative cell culture model system we intended to derive primary and immortalized cell cultures from various organs of *M*. *condylurus* bats. The aim of the study was to elucidate the suitability of primary and immortalized cells from different tissues for studying NPC1 receptor expression levels and their potential influence on EBOV replication. We investigated the NPC1 expression levels of different *M*. *condylurus* cells in comparison to two human cell lines and cells from another, phylogenetically distinct insectivorous bat, the European bat *Nyctalus noctula*. We found that the NPC1 receptor expression profile in *M*. *condylurus* as a potential EBOV reservoir is different to a highly symptomatic host such as humans. Further, we suggest a potential correlation between EBOV replication kinetics and the amount of expressed NPC1 in cells from different tissues.

## Materials and methods

### Animal handling and sample collection

*M*. *condylurus* bats were captured with mist nets at a residence in Koffikro Village in Ivory Coast (geographic coordinates: N 05° 19.340´; W 003° 49.431´). Subsequently, the bats were transported in cotton bags to LANADA Institute (Laboratoire National d’Appui au Développement Agricole) Bingerville, Ivory Coast. Organs were collected aseptically and put into 2 ml cryotubes with 1 ml Recovery Cell Culture Freezing Medium (12648010, Gibco). Immediately after the necropsy of each bat, the organs were frozen at -80°C (1°C/min) using a *CoolCell LX* (BCS-405, Biocision) and shipped to the Robert Koch Institute, Berlin, Germany using an IATA CryoShipper.

A single *Nyctalus noctula* bat was found dead in June 2017 in Berlin and presented to the Leibniz Institute for Zoo and Wildlife Research (IZW), Berlin, Germany. The bat was stored *in toto* at 4°C and provided for cell culture establishment to the Robert Koch Institute.

### Ethics statement

Animal work and necropsies were performed with the permission of the Laboratoire Central Vétérinair, Laboratoire National d'Appui au Développement Agricole (LANADA), Bingerville, Ivory Coast (No. 05/virology/2016). The animal care and use protocol adhered with the Ethics Committee of LANADA and National Ethics Committee for the Research (CNER). Consent existed to capture the bats from the owners of the residence in Koffikro Village. The bats were anaesthetized with Isoflurane (1214, cp-pharma) and euthanized by decapitation.

### Confirmation of bat species

The species of donor bats were determined by amplification and sequencing of a 241 bp fragment of the *cytochrome b* gene, commonly used for bat speciation [9, 10]. All organs and abbreviations used for this study are summarized in Table 1.

**Table 1:** List of bat organs used, primary cell cultures, cell lines and status of immortalization.

Species	Organ	Name of Primary Cell Culture	Days Until Passage 1	Spontaneous Immortalization	Name of SV40T Immortalized Cell Line
*M*. *condylurus*	Kidney	MoKi Prim	3	yes	MoKi*
* *	Testicles	MoTes Prim	14	yes	ND
* *	Lymph Node	MoLyN Prim	5	no	ND
* *	Brain	MoBra Prim	6	yes	ND
* *	Spleen	MoSp Prim	17	yes	ND
* *	Skin	MoSk Prim	14	yes	ND
* *	Muscle	MoMu Prim	1	yes	ND
* *	Heart	MoHe Prim	11	yes	ND
* *	Lung	MoLu Prim	11	yes	ND
* *	Trachea	MoTra Prim	17	yes	ND
* *	Liver	MoLi Prim	13	no	MoLi
* *	Bone Marrow	MoMac Prim	13	no	ND
*N*. *noctula*	Kidney	NyKi Prim	6	yes	NyKi

The first two letters indicate the species (Mo = *Mops condylurus*; Ny = *Nyctalus noctula*). The following two or three letters indicate the organ, from which the cell culture was derived from. SV40T immortalized cell lines have no further code. Prim = Primary cell cultures / not SV40T immortalized cell cultures.

* = cloned; ND = not done.

### Generation of NPC1 annotated contiguous sequences

As part of a *M*. *condylurus* transcriptome study (BioProject number: PRJNA506280) RNA was sequenced on a HiSeq1500 System (Illumina) and sequenced reads were assembled and annotated according to the Oyster River Protocol (MacManes, 2017). The contiguous sequences annotated as NPC1 were imported into Geneious to further examine potential reading frames and perform multiple alignments with NPC1 sequences from other species with ClustalW (S1A–S1C Fig). To select an antibody for the recognition of *M*. *condylurus* NPC1, we performed sequence alignments with human NPC1. Based on the similarity to human NPC1 we chose mouse anti-human NPC1 antibody (ab55706, abcam) targeting this region to measure NPC1 receptor expression levels.

### Generation of microbat primary cell cultures

All of the following steps to generate primary cell cultures (Prim) were performed under sterile conditions using a laminar flow hood. All organs were thawed at 37°C in a heat block and washed in a petri dish (94 x 15 mm, 452005, Brand) with phosphate buffered saline (PBS) containing 1x Antibiotic-Antimycotic (Gibco). After processing of the organ 5 x 10^5^ cells per well were seeded into a cell-culture treated six-well plate (734–0991, Nunc) (with the exception of MoTra Prim), which was then incubated in a humidified incubator with 5% CO_2_ at 37°C. Movement was avoided within the first 72 hours. After that, cells were inspected daily with medium changes on every second day. When cells reached 90% confluency they were expanded and finally frozen down for long-term storage in Fetal Bovine Serum (FBS) (1318D, Biochrom) with 10% dimethyl sulfoxide (DMSO) (Sigma). The establishment of bat primary cell cultures are described in detail in S1 Supporting Information.

### Immortalization with lentiviral vector and SV40T

MoKi Prim, MoLi Prim and NyKi Prim cells were immortalized by lentiviral transduction of SV40 large T antigen (G256-GVO-ABM, ABM) according to manufacturer’s instructions. Instead of using Polybrene we used ViraDuctin Lentivirus Transduction Kit (LTV-200, Cell Biolabs) for MoKi Prim and NyKi Prim cells and TransDux MAX Kit (LV860A-1, SBI) for the immortalization of MoLi Prim cells. All primary cells were infected for nine hours at a MOI of 1. Positive transgene expression was confirmed with confocal microscopy (S2 Fig) using SV40T Ag Antibody (sc-147, Santa Cruz Biotechnology) and goat anti-mouse IgG-Alexa Fluor 488 (115-545-003, Dianova).

The MoKi cell line acted as reference in this study for all flow cytometry experiments. To ensure that these cells have consistent and reproducible characteristics, the cell line was cloned. MoKi cells were passaged on a six-well plate (Nunc) and a single cell was isolated and cloned using an 8 mm PYREX Cloning Cylinder (09-552-21, Fisher Scientific).

### Confocal microscopy

For microscopy of MoTes Prim, MoLyN Prim, MoBra Prim, MoSp Prim, MoMu Prim and MoSk Prim cells, glass cover slips were coated with 0.01% rat tail collagen type I (C7661, Sigma-Aldrich) for one hour at room temperature. Before use they were washed twice with PBS.

Cells were grown overnight on glass or collagen-I-coated glass cover slips, washed and fixed for 15 min with 4% paraformaldehyde (0335.1, Carl Roth). Fixation was quenched with 0.3 M glycine in PBS, pH 7.4 for 15 minutes and three PBS washes. Cells were permeabilized for 30 min with 0.1% Triton X 100 (3051.3, Carl Roth) in PBS. Cells were washed with 0.1% Tween 20 (9127.1, Carl Roth) in PBS (PBST) and blocked for 30 min with 1% Albumin Fraction V (0163.2, Carl Roth) in PBST (Blocking solution). Cells were incubated at 4°C overnight with primary mouse monoclonal antibodies against NPC1 (ab55706, abcam) or mouse IgG2a kappa light chain isotype control antibodies (NB600-986, Novus Biologicals), both diluted 1:800 in blocking solution. For actin filament staining, a 100 nM solution of Acti-stain 555 Phalloidin (PHDH1-A, Cytoskeleton) was used for 30 min. After intensive washing, cells were incubated for 60 min with donkey anti-mouse IgG H&L Alexa Fluor647 (ab150111, abcam) as secondary antibody diluted 1:1000 in blocking solution. For mounting of samples with stained actin cytoskeleton ddH_2_O was used, while all other samples were mounted in ROTI Mount FluorCare DAPI (HP20.1, Carl Roth) for antifading properties and DAPI counterstaining. A confocal laser scanning microscope (Zeiss LSM 780) was used to visualize the different cell morphologies. For comparison of NPC1 expression levels, 20 different random fields on each coverslip were analyzed and identical microscope settings were used for all samples.

### Flow cytometry

For each cell type one million cells were transferred to one well of a V-bottom 96-well microplate. Three samples were analyzed with Beckman Coulter Cytoflex S for each cell population and the whole experiment was performed twice for all cells. Cells were pelleted at 500 g for 3 min, washed with PBS and stained with Live/Dead Fixable Violet Dead Cell Stain Kit (L34964, Thermo Fisher). The reconstituted fluorescent reactive dye was diluted 1:100 in PBS, 100 μl per well were added and incubated 30 min at 4°C in the dark. Afterwards cells were washed with 2% FBS in PBS and fixed for 20 min with 4% paraformaldehyde at 4°C. Cells were washed twice with PBS and permeabilized for 30 min with 0.1% Triton X 100 in PBS at room temperature. After blocking for 30 min with blocking solution at room temperature, cells were incubated at 4°C overnight with primary mouse monoclonal antibodies against NPC1 or mouse IgG2a kappa light chain isotype control antibodies, both diluted 1:100 in blocking solution. Cells were washed twice with PBST and then incubated for 60 min at room temperature with donkey anti-mouse IgG H&L Alexa Fluor647 as secondary antibody diluted 1:250 in blocking solution. Finally cells were washed twice with PBST and were resuspended in PBS containing 2% FBS, 1mM EDTA, 0.1% sodium azide, pH 7.4 (FACS buffer).

Forty thousand events were collected per run. Five primary cell cultures were not tested using flow cytometry (MoHe Prim, MoKi Prim in low passage number, MoMac Prim, MoLyN Prim and NyLi Prim), because the minimum cell number per assay of 5 x 10^6^ cells could not be achieved. The detectable intracellular antibody staining was significantly higher than the detectable binding of the isotype control in all cells (S3 Fig).

Results are presented as fold-change and the non-specific background of the isotype control was subtracted from the mean fluorescence intensity. For comparisons of the NPC1 expression levels, the MoKi cell line was set to 100% and the expression levels of the other cells were referred to the MoKi cell line. Deviations of more than 20% from the arithmetical average within the triplicate were not accepted and these data were not taken into consideration.

### Infection of cells and measurement of viral RNA

Infectious work with EBOV was performed in the BSL4 facility of the Rocky Mountain Laboratories according to standard operating protocols (SOPs) approved by the Institutional Biosafety Committee. Four representative primary and immortalized cell cultures from *M*. *condylurus* (MoTra Prim, MoSp Prim, MoHe Prim and MoKi) were seeded in six-well plates and infected with EBOV (strain Makona, C07) with a MOI of 0.1 for 1 hour. Cells were washed twice with 3 ml cell culture medium (DMEM, 15% FBS, 1 mM L-glutamine, 50 U/ml penicillin, 50 μg/ml streptomycin). Three ml cell culture medium was added to the cells. 250 μl supernatant per well was collected after 1, 24, 48, 72 and 96 hours in AVL Buffer (19073, Qiagen). Samples were mixed with an equal volume of absolute ethanol before removing from BSL4 according to SOPs. Viral RNA was extracted using the QIAamp Kit (Qiagen) according to the manufacturer’s instructions. Viral RNAs were quantified with qRT-PCR (Applied Biosystems 7500) using the AgPath-ID One-Step RT-PCR Kit (4387391, Thermo Fisher). Primers/probe targeted EBOV VP30 (forward: ACTCCTACTAATCgCCCgTAAg; reverse: ATCAgCCgTTggATTTgCT; probe: FAM-CACCCAAggACTCgC-MGB). For each PCR reaction, 3 μl of the RNA sample was added to 22 μl master mix containing: 400 nM forward/reverse primers and 200 nM probe, 1 μl detection enhancer, 1x RT-PCR buffer and 1x RT-PCR enzyme mix. Samples were incubated for 15 min at 45°C, 10 min at 95°C followed by 45 cycles of 15 sec at 95°C and 45 sec at 60°C. Measured CT values were compared to a standard curve, which was produced using EBOV *in vitro* transcripts (concentrations ranging from 10−10^7^ copies) to determine the viral copy number of each sample.

## Results

### Establishment and characterization of microbat primary cell cultures

Primary cell cultures from 12 different organs/tissues from *M*. *condylurus* (kidney, testicle, lymph node, brain, spleen, skin, muscle, heart, lung, trachea, liver, bone marrow) and one organ from *N*. *noctula* (kidney) were established (Table 1). The point in time when the primary cells reached confluency and were passaged for the first time varied depending on the organ, e.g. muscle cells reached confluency after one day, whereas spleen and trachea cells reached confluency after 17 days (Table 1).

Primary cell cultures were predominantly heterogeneous with a broad variety of different cell morphologies (S4A Fig). The shape and size of the cells varied from an epitheliod morphology, e.g. in kidney cell cultures, to a fibroblastic morphology, e.g. in lung cell culture. Noticeable cell types were bone marrow-derived macrophages (BMDMs) with a multivesiculated morphology, neuronal cells with dendrites in brain cell cultures, polymorphonuclear cells from lymph nodes or myoblasts forming polynuclear syncytia in muscle cell cultures (S4C–S4F Fig). Most primary cell cultures from *M*. *condylurus* underwent a spontaneous immortalization process, except for liver, bone marrow and lymph node. For the majority of the primary cell cultures, the growth rate did not slow down after 15 passages. Six of these cell cultures were passaged 35–70 times without showing changes in growth rates (Table 1). Continuous passaging resulted in a decrease in cell variety and in a relatively homogenous cell culture (S4G and S4H Fig). Other cell cultures were homogenous from initial passages such as lung, trachea, skin and muscle cell cultures from *M*. *condylurus* and the kidney cell culture from *N*. *noctula* (S4B Fig).

### SV40T-induced immortalization of kidney and liver cells

Primary kidney and liver cells from *M*. *condylurus* and primary kidney cells from *N*. *noctula* were immortalized with SV40T (Table 1). The expression of the SV40 large T antigen was confirmed with immunofluorescence staining and subsequent confocal microscopy (S2 Fig). For the MoKi cell line we cloned a cell type with epithelioid morphology. No morphological differences between the SV40T immortalized cells and the corresponding primary cell type were observed. Comparing MoLi cells in passage four to MoLi cells in passage 11 after immortalization, clear morphological changes were visible (S2 Fig), which included a reduction in cell size while no remaining primary cell types were recognizable.

### *M*. *condylurus* NPC1 sequence shares homology with human NPC1

The partial NPC1 sequence identified from the *M*. *condylurus* transcriptome corresponded to amino acids 105–605 of human NPC1. We implemented this sequence information to select a human NPC1 antibody targeting an immunogenic and conserved region of the molecule. We selected an antibody targeting the region between amino acids 151–250, which possessed 91% identity between *M*. *condylurus* and the human sequence (S1A and S1B Fig).

Recently it was shown that a single amino acid change, D502F, in a central region of NPC1 domain C strongly hinders EBOV-GP-NPC1 binding [36]. An amino acid sequence alignment of *M*. *condylurus* NPC1 with other relevant species revealed that *M*. *condylurus*, human and other potential reservoir species like *H*. *monstrosus* encode for an aspartic acid at position 502 (S1C Fig).

### NPC1 receptor expression levels vary between cell type and passage number

#### Confocal microscopy

Comparison of the immunofluorescence signal intensities of 22 primary and immortalized cell cultures derived from different organs and tissues elucidated notable variations in NPC1 receptor expression levels. According to the strength of the immunofluorescence signal, cell cultures were classified as expressing high (dark red), moderate (medium red) or low (light red) NPC1 levels (Table 2).

**Table 2:** Cells tested for their NPC1 receptor expression levels with confocal microscopy and flow cytometry.

Organ	Passage No.	Status of Immortalization*	Cell Culture Name	Composition of Cell Culture	NPC1 Receptor Expression Levels	
					Confocal Microscopy	Flow Cytometry in [%] compared to MoKi cells
Cervix	5	I	HeLa	Homogenous	**HIGH**	n.a.
Kidney	29	I	NyKi	Homogenous		239,83
Kidney	4	P	NyKi Prim.	Heterogenous		213,66
Spleen	29	P	MoSp Prim. Late	Homogenous		207,49
Kidney	5	I	HEK293	Homogenous		198,37
Muscle	1	P	MoMu Prim.	Homogenous		143,81
Brain	1	P	MoBra Prim.	Heterogenous	**MODERATE**	122,44
Liver	5	P	MoLi Prim.	Heterogenous		n.a.
Liver	14	I	MoLi	Homogenous		109,04
Kidney	9	I	MoKi	Homogenous		100
Heart	65	P	MoHe Prim. Late	Homogenous		85,07
Testicles	1	P	MoTes Prim.	Heterogenous		74,52
Kidney	19	P	MoKi Prim. Late	Homogenous		52,71
Bone Marrow	1	P	MoMac Prim.	Homogenous		n.a.
Heart	5	P	MoHe Prim.	Heterogenous		n.a.
Lymph Node	1	P	MoLyN Prim.	Heterogenous		n.a.
Kidney	7	P	MoKi Prim.	Heterogenous	**LOW**	n.a.
Skin	1	P	MoSk Prim.	Heterogenous		79,37
Lung	7	P	MoLu Prim.	Homogenous		71,90
Liver	7	P	NyLi Prim.	Heterogenous		n.a.
Spleen	5	P	MoSp Prim.	Heterogenous		n.a.
Trachea	5	P	MoTra Prim.	Homogenous		50,55

Status of immortalization: I = SV40T immortalized; P = Primary cells or spontaneously immortalized cells.

**Fig 1:**
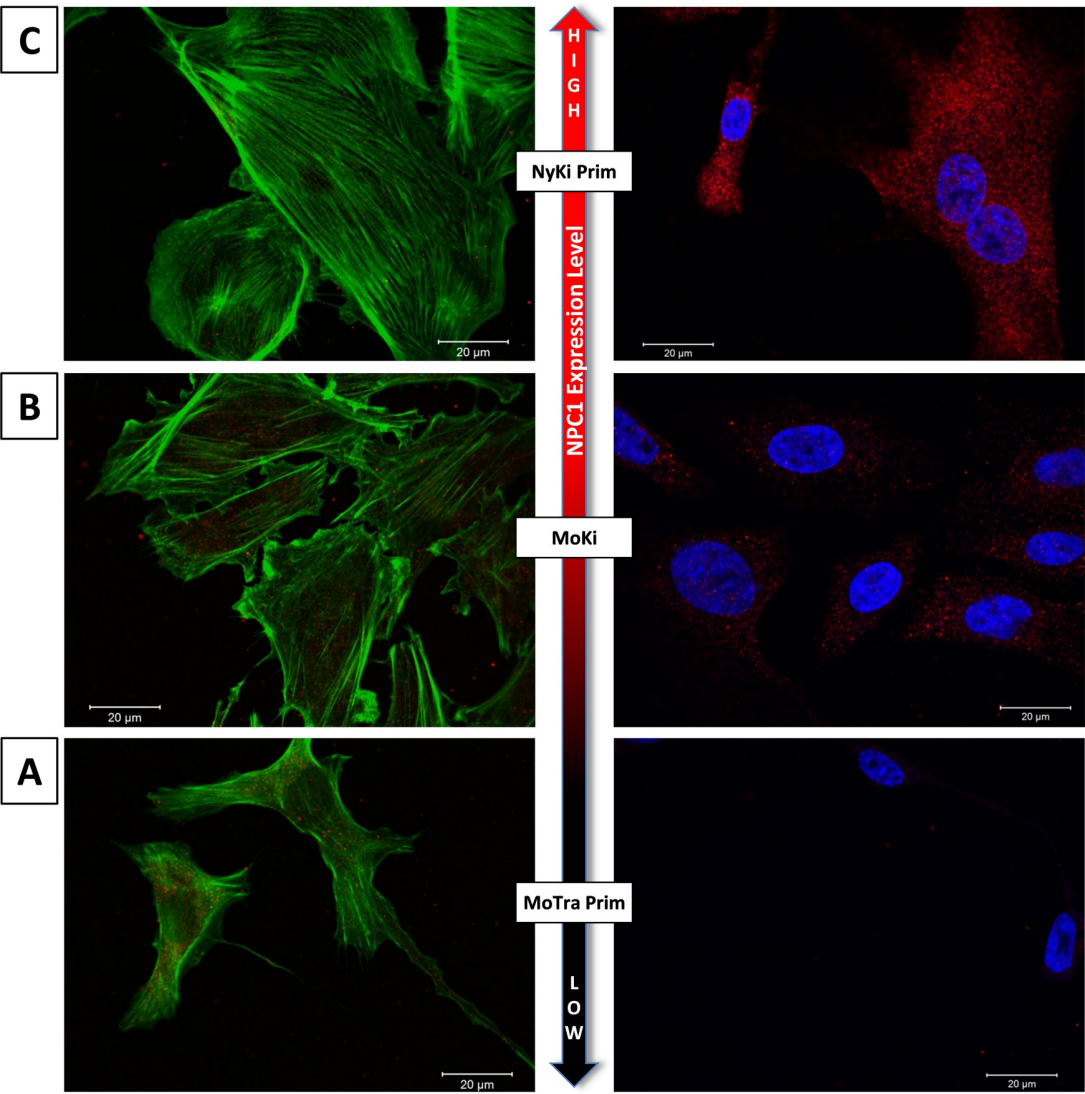
Comparison of NPC1 receptor expression levels in different bat cells using confocal microscopy. Left column: stained actin filaments (green). Right column: stained NPC1 receptor (red) and cell nuclei (blue). NPC1 receptor expression levels: A) MoTra Prim (low), B) MoKi (moderate), C) NyKi Prim (high).

**Fig 2:**
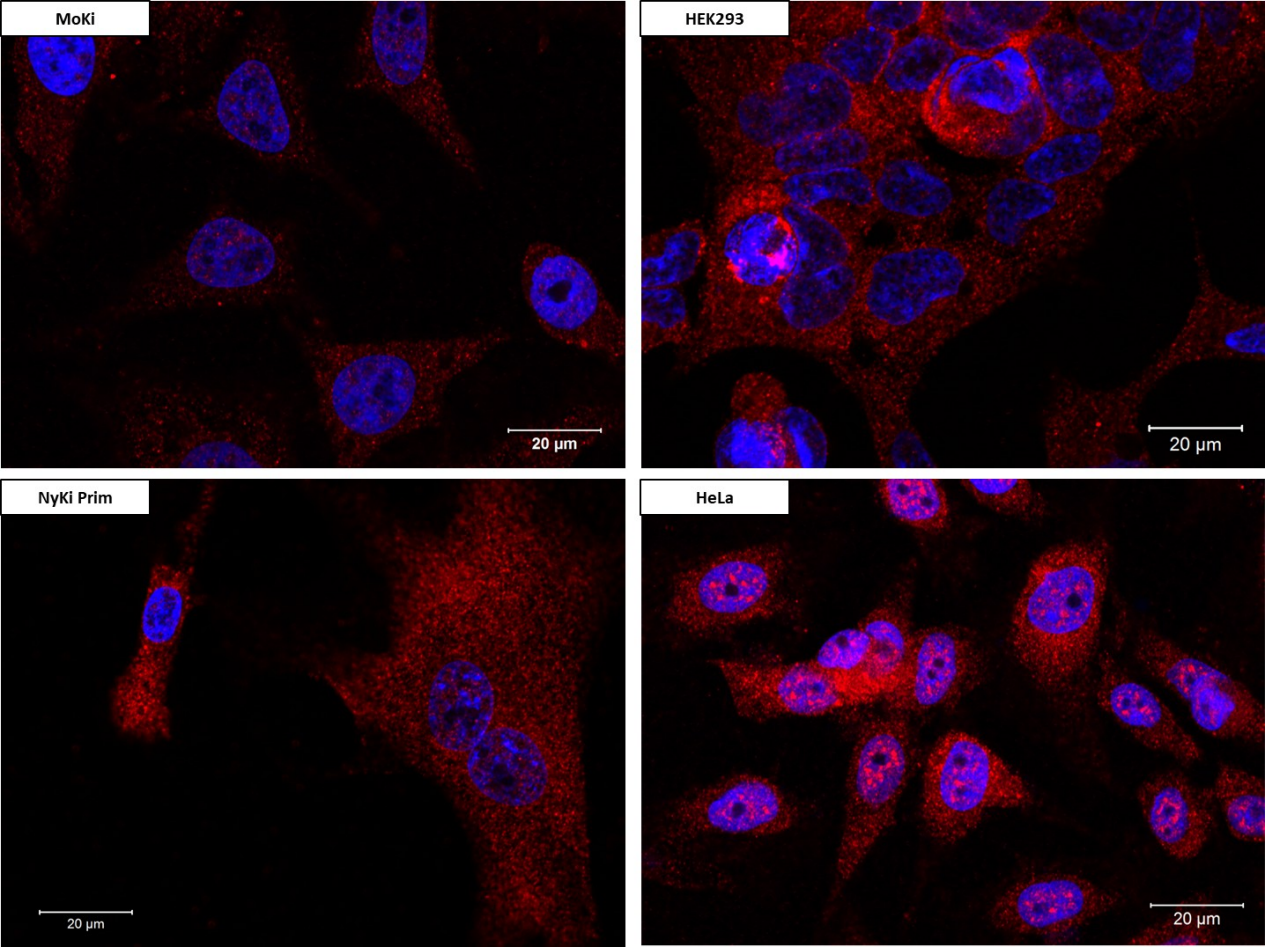
Comparison of NPC1 receptor expression levels between two human cell lines and two different bat cell cultures. Stained NPC1 receptor (red), cell nuclei (blue). NPC1 receptor expression levels of NyKi Prim, HEK293 and HeLa cells (high), MoKi cells (moderate).

Examples of the cultures classified as low, medium and high are shown with confocal microscopy images in Fig 1. All cells but one (MoSp Prim Late cells) derived from *M*. *condylurus* had lower NPC1 expression levels than the tested human cells and cells from the European bat *N*. *noctula*. The lowest levels of NPC1 expression were detected in MoTra Prim and MoSp Prim cells (Fig 1A). Moderate expression levels could be detected in MoHe Prim cells and MoKi cell line (Fig 1B). The highest NPC1 expression levels were measured in HeLa cells, HEK293 cells and NyKi Prim cells (Fig 1C, Fig 2).

Comparison of primary cells with low passage number to those with higher passage number revealed that NPC1 expression levels increased with passage number. This was evident for MoSp Prim, MoHe Prim and MoKi Prim cell cultures with high passage numbers, which showed higher NPC1 expression levels than the corresponding cultures with low passage numbers. This difference was particularly distinct for MoSp Prim cells, where low passage number MoSp Prim cell cultures (together with MoTra Prim cells) displayed the lowest expression levels of all tested cells, whereas high passage MoSp Prim cell cultures revealed the highest NPC1 expression levels of all *M*. *condylurus* cells tested (S5 Fig).

In contrast to primary cell cultures, the NPC1 expression in immortalized cultures gave disparate results depending upon the cell type under investigation. For MoLi Prim and immortalized MoLi cells the NPC1 expression was similar. For *M*. *condylurus* kidney cells, NPC1 expression level in immortalized cells was similar to those observed for high passage number cultures, but overtly higher than in low passage number cells of the same organ. In contrast, immortalized NyKi cells revealed a lower NPC1 expression level than the corresponding NyKi Prim cells (Table 2).

#### Flow cytometry

**Fig 3:**
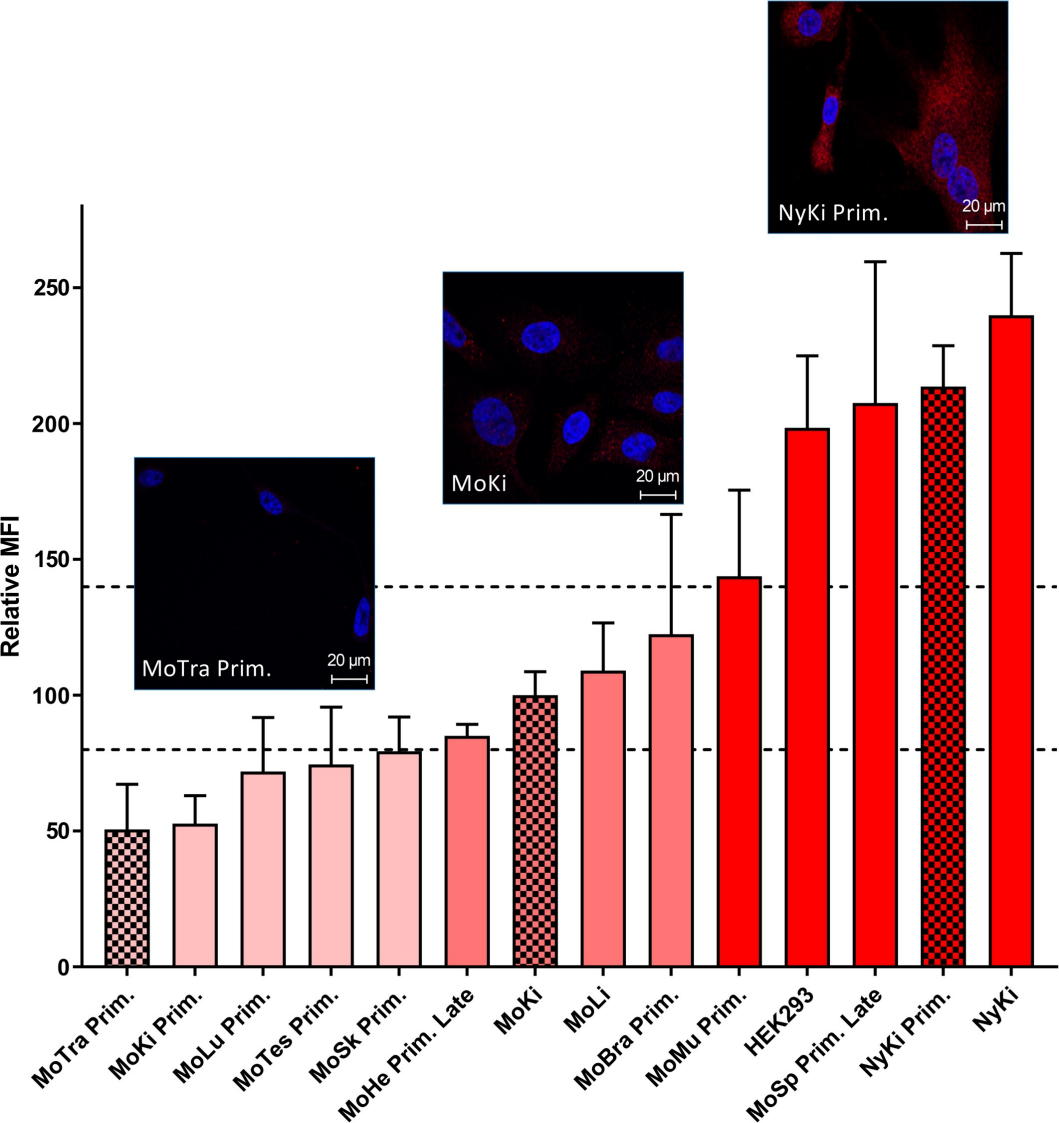
Percentage of NPC1 expression levels in comparison to MoKi cell line determined with flow cytometry. For comparisons of NPC1 expression levels in different cell cultures, the mean fluorescence intensity (MFI) value of the MoKi cell line was set to 100% and the expression levels of the other cells were referred to the MoKi cell line. NPC1 receptor expression levels: Low expression level (light red: below 80%), moderate expression level (medium red: 80–140%), high expression level (dark red: over 140%). Confocal pictures of three representatives for different NPC1 expression levels: MoTra Prim (low), MoKi (moderate), NyKi Prim (high).

Measurement of NPC1 mean fluorescence intensities of 14 cell cultures (Table 2) identified significant variations in NPC1 receptor expression levels between cell types. Relative to immortalized MoKi cells, cell cultures were classified into high (> 140%), moderate (80–140%) or low (< 80%) according to their NPC1 expression levels (Fig 3). Ten of 11 cell cultures derived from *M*. *condylurus* showed lower NPC1 expression than detected in human cells (HEK293) or in cells from *N*. *noctula* (NyKi and NyKi Prim), respectively. The lowest level of NPC1 was detected in MoTra Prim cells. Moderate expression levels were detected in MoHe Prim Late cells and MoKi cells. The highest NPC1 expression levels were observed in HEK293, NyKi Prim and NyKi cells. A noticeable high NPC1 expression level was detected in MoSp Prim Late cells.

The ranking into high, moderate and low NPC1 expression based on flow cytometry measurements corresponded with similar observations made with confocal microscopy except for MoTes Prim and MoKi Prim Late cultures, which had a higher NPC1 expression level detected in microscopy than flow cytometry. Depending on the heterogeneity of the cell culture the acquisition of data was performed in generous gates to measure a large variety of different cell types (Table 2). MoTra Prim were very homogenous (small gates) while we could see a large variety of cell types in MoLi Prim cell cultures (big gates) (S6 Fig). Triplicate measurements of MoLi Prim cells were highly variable and their fluorescence intensity was not included in the analysis. Instead we immortalized liver cells for inclusion of MoLi cells into the analysis. Immortalization and continuous passaging resulted in relative homogenous cell populations compared to primary liver cell cultures (S7 Fig).

#### Infection of cell cultures from *M*. *condylurus* with Ebola virus (EBOV)

**Fig 4:**
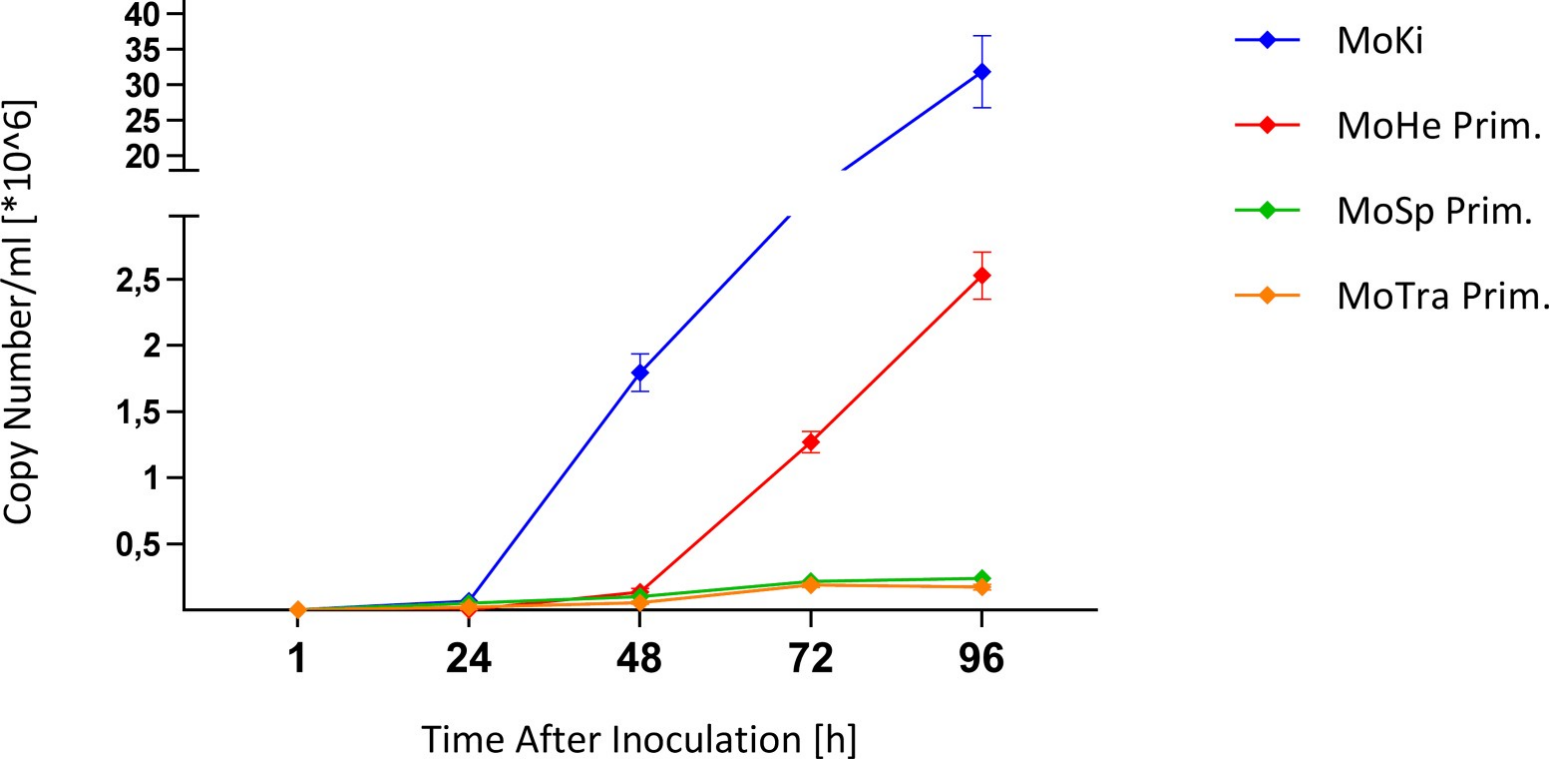
Ebola virus (EBOV) replication kinetics in different *M*. *condylurus* cells. Viral genome copy numbers/ml of infected cell cultures from *M*. *condylurus* (MoTra Prim, MoSp Prim, MoHe Prim and MoKi) were determined by qRT-PCR. Different levels of EBOV replication: low virus replication in MoTra Prim (orange) and MoSp Prim cells (green), moderate virus replication in MoHe Prim cells (red), high virus replication in MoKi cells (blue).

For infection experiments with EBOV we selected three primary and one immortalized *M*. *condylurus* cell types with a spectrum of NPC1 expression levels: MoTra Prim and MoSp Prim cells represented low NPC1 expression; MoHe Prim and MoKi cells represented moderate NPC1 expression, with clearly higher NPC1 expression in MoKi cells (Table 2). Cells were infected with EBOV Makona C07 (MOI 0.1) and cell culture supernatants were collected daily for 4 days. Infected MoKi cells displayed the highest viral genome copy number with more than 3.1 x 10^7 copies/ml (Fig 4). They revealed a more than ten-fold higher viral genome copy number than MoHe Prim cells after exponential growth until 96 hours post infection. In both cell cultures with low NPC1 receptor expression levels, less than 2.5 x 10^5 viral genome copies/ml were detected.

## Discussion

Differences in the NPC1 receptor expression level of cell cultures derived from human, *M*. *condylurus* and *N*. *noctula*, a phylogenetically distant European bat unlikely to be a reservoir host of ebolaviruses, were observed in this study. Expression levels of NPC1 in cell cultures derived from different tissues of *M*. *condylurus* varied between primary and immortalized cell types. Finally, we suggest a potential correlation between NPC1 receptor expression level and EBOV replication kinetics.

Recently it was shown that polymorphisms in the NPC1 domain C strongly influenced EBOV-GP-NPC1 binding where the NPC1 sequence of the African straw-colored fruit bat *E*. *helvum* containing a phenylalanine at amino acid 502 imparted diminished susceptibility to EBOV infection [36]. The amino acid sequence alignment of *M*. *condylurus* NPC1 with other tested species (S1C Fig) revealed that *M*. *condylurus* cells, human cells and cells from other mammals have an aspartic acid at position 502. *M*. *condylurus* has no D502F protein sequence variation like *E*. *helvum*, suggesting the interaction between EBOV-GP and *M*. *condylurus* NPC1 would be functional, imparting susceptibility to EBOV infection. Aside from NPC1 polymorphisms, we hypothesize NPC1 abundance in the endosomes and lysosomes could also affect susceptibility to EBOV infection by influencing viral entry and have implications for tissue tropism.

We detected significant variations in the NPC1 receptor expression levels in 22 tested different cell cultures. According to the strength of the immunofluorescence signal, cells were categorized into groups with high, moderate or low NPC1 expression. Our findings revealed lower NPC1 expression levels in numerous *M*. *condylurus* cell cultures when compared to two human and one European bat cell type. However in comparison with the proteomic database [37] the amount of expressed NPC1 in HeLa or HEK293 cells is lower than in 32 other human cell lines such as A549 or Hep G2 cells (S8 Fig). When considering the ranking of NPC1 expression levels in human tissues we could observe some overt differences compared to *M*. condylurus cells: According to different databases [37–40] the NPC1 expression level in human testis is high compared to other human tissues. In contrast, the NPC1 expression level in *M*. *condylurus* testis was low compared to other tested tissues from this bat species. Conversely, the NPC1 expression level in the human brain is very low [37], while we could detect relatively high NPC1 expression levels in *M*. *condylurus* brain cells compared to other tested tissues from this bat species.

We observed the lowest NPC1 expression levels in MoTra Prim and MoSp Prim cells which correlated with low levels of viral genome copies following EBOV infection. These findings suggested viral entry was impaired. Only a few cell types have been described to be refractory to EBOVs infection. Nearly all human cell types and a very broad range of other mammalian cells are susceptible to filovirus infection. Those that are refractory are mosquito cells, cells of lymphoid origin including human B-, T- and NK cells and murine lymphoid cell lines [25, 44, 50]. Although NPC1 is expressed in T cells to relatively high amounts [37], these cells are resistant to EBOV infection *in vitro*. This is indicating that NPC1 is an important but not the sole factor for EBOV infection on cellular level. Recent studies have shown that EBOV directly binds to T lymphocytes without causing infection, induces T cell death and is hereby contributing to lymphopenia [51, 52]. To our knowledge no other mammalian cell type apart from cells of lymphoid origin, originating from NPC1 diseased patients or with the D502F polymorphism has been shown to be refractory to EBOV infection.

In contrast to low EBOV genome copy numbers in bat trachea and spleen cells, we observed moderate and high genome copy numbers in MoHe Prim cells and MoKi cells, respectively. Both cell types were classified to have a moderate level of NPC1 expression. According to our confocal and flow cytometry data, MoKi cells as an immortalized cell type showed the highest NPC1 expression level of all infected cells and also exhibited the highest EBOV replication rate. Previous studies have ascertained that cells from a wide variety of bat species support filovirus replication *in vitro* [41, 45] leading generally to high virus titers. All previously used cells were immortalized cell cultures, suggesting a potential inaccuracy compared to their corresponding primary cells resulting in a misinterpretation as shown for EBOV replication in R06 cells and Rousettus bats [46, 53, 54]. The noticeable lower NPC1 expression levels in most tested cells from *M*. *condylurus* compared to human cells (Fig 2) potentially leads to lower levels of virus replication in *M*. *condylurus* cells. This is underlined by the evidence of two cell lines almost resistant to EBOV replication showing the lowest NPC1 expression levels of all tested cells. These are promising results for further *in vitro* infection experiments in *M*. *condylurus* cells, to prove whether NPC1 expression levels correlate with the replication of infectious EBOV particles in primary cells.

Several fundamentally different mechanisms to move sterols within cells through transport vesicles, carrier-mediated diffusion or direct contacts between two membranes operate simultaneously [55, 56]. Although mechanisms of intracellular cholesterol trafficking in bats still remain unstudied, it is conceivable that the exceptional low NPC1 expression levels in many cell types of *M*. *condylurus* are sufficient to fulfil all functions in the pathways of intracellular cholesterol trafficking so that the bats are not showing any disorder related to Niemann-Pick C disease. Because NPC1 is an evolutionary very conserved protein, which can even be found in primitive eukaryotic organisms like green algae, fungi or insects [57, 58]; (S1B Fig) it would be implausible that an alternative cholesterol transport protein would have evolved in bats. *M*. *condylurus* is known to have a pronounced heat tolerance [59]. These bats use high-temperature roosts and show high body temperatures of 43°C at 45°C [60]. Highly recurrent exposure to high body temperatures presumably also leads to particular cellular adaptations in *M*. *condylurus* like changes in the amount of cholesterol in cellular membranes. How the suspected temperature adaptations are influencing the NPC1 expression levels and how high ambient temperatures might influence the course of infection with EBOV needs to be addressed in further investigations.

The susceptibility of immortalized cell lines does not necessarily inform about the host range and tissue tropism of a virus, as demonstrated by past studies showing that EBOV replication in the R06E cell line [46], an immortalized fibroblast cell line from *R*. *aegyptiacus* [61], did not correspond with significant viral replication in fruit bats after experimental inoculations [41, 53, 54]. A disadvantage of immortalized cell lines is the genetical and phenotypical difference from their *in vivo* counterparts, while primary cells maintain many of the important markers and functions seen *in vivo* [62–64]. The uniqueness of our study includes the use of primary cell cultures at low passage to closely mimic cell types present in their respective organs. The biological properties and the genetic background of reservoir-derived primary cells are in contrast to immortalized cells closer to the *in vivo* situation [63] and are an advantageous tool for *in vitro* studies on virus-host interactions. Continuous passaging of primary cells led to a loss of cell diversity, probably through the selection processes due to the cell culture conditions. We have shown that the morphology and the general characteristics including the NPC1 expression levels of the cells were also changing dramatically after passaging. The majority of MoSp Prim cells with a low passage number showed a very low NPC1 expression level. Through passaging an infrequent cell type with a differing morphology and high NPC1 expression level was enriched. To acquire sufficient cell numbers for measurements with flow cytometry, MoSp Prim needed two additional passages. Within these passages the composition of the primary cell culture changed and cells with high NPC1 expression levels became dominant. These results reflect the possible dramatic change of the characteristics of cell cultures within a very short time. Although the vast majority of cell culture experiments in filovirus research, including model systems for entry and replication, are performed with immortalized cell lines [36, 41–49], the limitations of these model systems should be taken into consideration. We have also demonstrated the importance of the use of cells in low passage numbers not taken into consideration in many studies. Passaging, immortalization and cloning lead to significantly changed cells with acquired features that are not characteristic for the related cells in the organ they were derived from [45].

While spontaneous immortalization of cell cultures is an extremely rare event in human and avian cells, rodent and rainbow trout cells immortalize spontaneously at much greater rates [65–68]. For bat cells, spontaneous immortalization also seems to be a rare event. One example is the lung-derived CpLu cell line from *Carollia perspicillata* [69]. It was reported that no bat primary cell culture, established from 20 different organs of *P*. *alecto*, immortalized spontaneously [70]. In this regard it was an interesting observation that most primary cell cultures from *M*. *condylurus* underwent a spontaneous immortalization process, proven by passaging six cultures more than 35 times without any decrease in growth rate. Spontaneous immortalization is usually a rare event requiring genomic instability, such as alterations in chromosomes and mutations in genes [71]. Whether *M*. *condylurus* cells are genetically unstable and acquire mutations easily, have tissue-resident somatic stem-cell populations giving rise to the cell lines [67, 72] or have differences in telomerase activities or particular cell cycle regulations has to be addressed in future studies.

In summary NPC1 expression levels differ significantly in cells derived from distinct organs of *M*. *condylurus*, with high variations observed in NPC1 expression within a bat species. We discovered key factors that influence NPC1 expression levels in cell culture including passage number and immortalization processes. According to NPC1 expression, low, moderate and high cell cultures were able to be delineated, which upon infection with EBOV seemed to reveal a potential correlation between NPC1 expression levels and virus titers. Different amounts of infected cells of the used cell cultures might have affected the results. Also, various other cellular factors may influence EBOV replication efficacy. NPC1 knock down or knockout experiments may be useful to approach some of these difficulties.

*M*. *condylurus* cell cultures typically displayed low NPC1 expression levels and limited EBOV replication in those cultures suggesting the respective tissues *in vivo* would be poorly susceptible to EBOV infection. We hypothesize that low NPC1 expression would facilitate viral persistence in the host by imparting a divergent tissue tropism, one that leads to asymptomatic infection profiles. Our findings convey that studying primary cells are advantageous over immortalized cells and that receptor expression levels and not only sequence information of entry receptors should be investigated when identifying reservoir hosts.

Future infection experiments with a variety of different *M*. *condylurus* cells could reveal, if these cells react differently to filovirus infection than other bat or human cell lines. We expect that cells from an ebolavirus reservoir species support virus replication without destruction of infected cells and possible virus persistence. Nevertheless, the NPC1 receptor is not the sole component of the filovirus entry process and the complex interplay with all components of the immune system cannot be tested with *in vitro* infection experiments. In order to address the limitations of the study by using cell cultures only, attempts to detect and isolate EBOV in M. *condylurus* have to be enforced, allowing the determination of the tissue tropism of EBOV. If not found in naturally infected animals, experimental infections of *M*. *condylurus* bats with EBOV might be inevitable to determine the role of these bats for the ecology of ebolaviruses.

## Supporting information

S1 Fig *M*. *condylurus* NPC1 sequence alignments. A: Alignment of immunogenic region (amino acids 151–250) for mouse anti-human NPC1 antibody (ab55706, abcam) of human (1) and *M*. *condylurus* NPC1 (2). B: NPC1 sequence homology (immunogenic region) between different taxa. C: Multiple alignment of NPC1 partial sequence, domain C (amino acids 491–517). Residue 502 is highlighted with a red box. Highly conserved amino acids are shown in black. Less conserved amino acids in dark and light grey. Other sequences than *M*. *condylurus* NPC1 are publicly available or from Ng et al. [36].
(TIF)

S2 Fig SV40T expression in MoLi cell line. SV40T expression (magenta) was confirmed with confocal microscopy (63x); stained actin filaments (green). Immortalized liver cells with different passage numbers–A: passage four (MoLi Early); B: passage 11 (MoLi Late). The red arrows show primary liver cells without SV40T expression in passage four after immortalization.
(TIF)

S3 Fig Flow cytometry gating strategy (MoKi cell line reference). A: Living cells (P1, green); B: singlets (P2, orange); C: Living singlets (orange) in total MoKi cell population; D: relative fluorescence after NPC1 staining (MoKi-A3,B3,C3; red), isotype control (blue).
(TIF)

S4 Fig Phase contrast microscopy of *M*. *condylurus* primary cells. A: Heterogenous liver cell culture with a broad variety of cell types (MoLi Prim); B: Homogenous trachea cell culture with only one recognizable cell type (MoTra Prim); Noticeable cell types–C: Bone marrow-derived macrophages (MoMac Prim); D: Brain cells (MoBra Prim); E: Polymorphonuclear cells (MoLyN Prim); F: Polynuclear syncythia (MoMu Prim); Primary kidney cells with different passage numbers–G: passage one (MoKi Prim Early); H: passage 32, decrease in cell variety (MoKi Prim Late); C: 20x, all other: 40x.
(TIF)

S5 Fig Comparison of NPC1 receptor expression levels in MoSp Prim cells with different passage numbers using confocal microscopy. Left column: stained actin filaments (green); Right column: stained NPC1 (red), nucleus stained with DAPI (blue). A: Primary spleen cells in passage 5 (MoSp Prim Early) with low NPC1 expression; B: Primary spleen cells in passage 29 (MoSp Prim Late) with high NPC1 expression. A+B: 63x.
(TIF)

S6 Fig Comparison of homogenous and heterogenous cell culture with flow cytometry. A: homogenous cell culture (MoTra Prim); B: heterogenous cell culture (MoLi Prim), large variety of cell types.
(TIF)

S7 Fig Comparison of primary and SV40T immortalized liver cells with flow cytometry. A: large variety of cell types in primary liver cells (MoLi Prim.); B: SV40T immortalized liver cells (MoLi). Immortalization and continuous passaging resulted in relative homogenous cell populations compared to primary liver cell cultures.
(TIF)

S8 Fig NPC1 receptor expression levels in different human cell lines. The amount of expressed NPC1 in HeLa and HEK293 cells (red box) is lower than in several other human cell lines. Protein expression is shown as log10 normalized iBAQ intensity; data available on *Proteomics DB* [37].
(TIF)

S1 Supporting Information Establishment of bat primary cell cultures. (DOCX)
