# Supplementary figures and images for: Utility of primary cells to examine NPC1 receptor expression in Mops condylurus, a potential Ebola virus reservoir
Source: PLoS Negl Trop Dis. 2020 Jan 21;14(1):e0007952. doi: 10.1371/journal.pntd.0007952 (PMC6994141; doi:10.1371/journal.pntd.0007952)

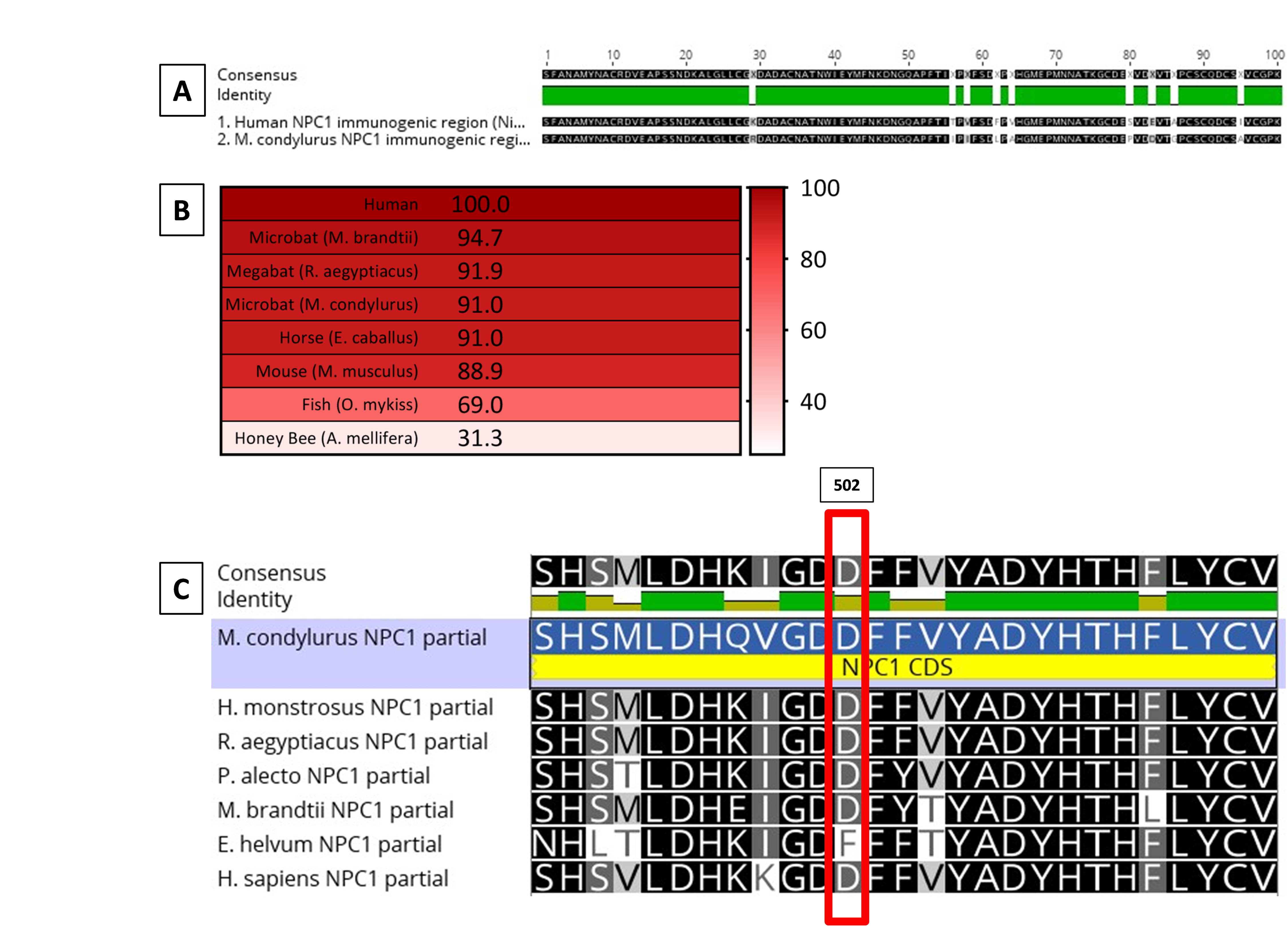

Supplement: S1 Fig — A: Alignment of immunogenic region (amino acids 151–250) for mouse anti-human NPC1 antibody (ab55706, abcam) of human (1) and M. condylurus NPC1 (2). B: NPC1 sequence homology (immunogenic region) between different taxa. C: Multiple alignment of NPC1 partial sequence, domain C (amino acids 491–517). Residue 502 is highlighted with a red box. Highly conserved amino acids are shown in black. Less conserved amino acids in dark and light grey. Other sequences than M. condylurus NPC1 are publicly available or from Ng et al. [36]. (TIF) [file pntd.0007952.s001.tif]

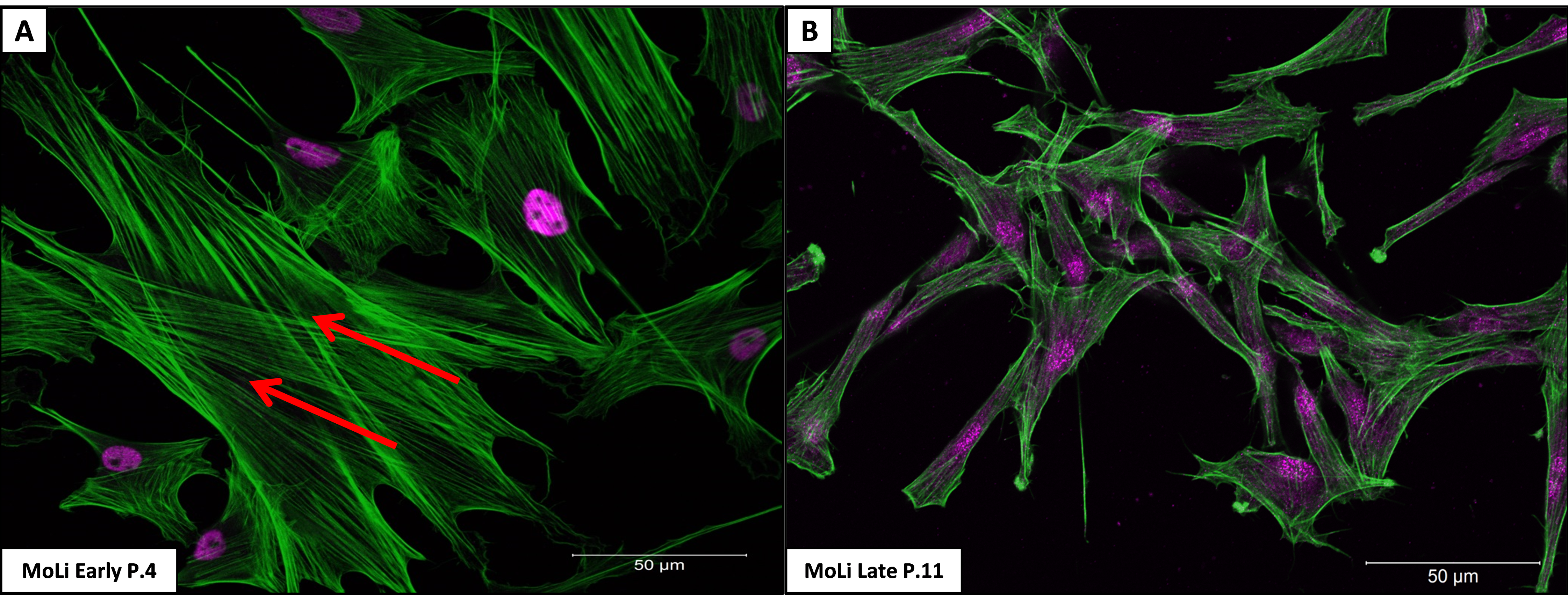

Supplement: S2 Fig — SV40T expression (magenta) was confirmed with confocal microscopy (63x); stained actin filaments (green). Immortalized liver cells with different passage numbers–A: passage four (MoLi Early); B: passage 11 (MoLi Late). The red arrows show primary liver cells without SV40T expression in passage four after immortalization. (TIF) [file pntd.0007952.s002.tif]

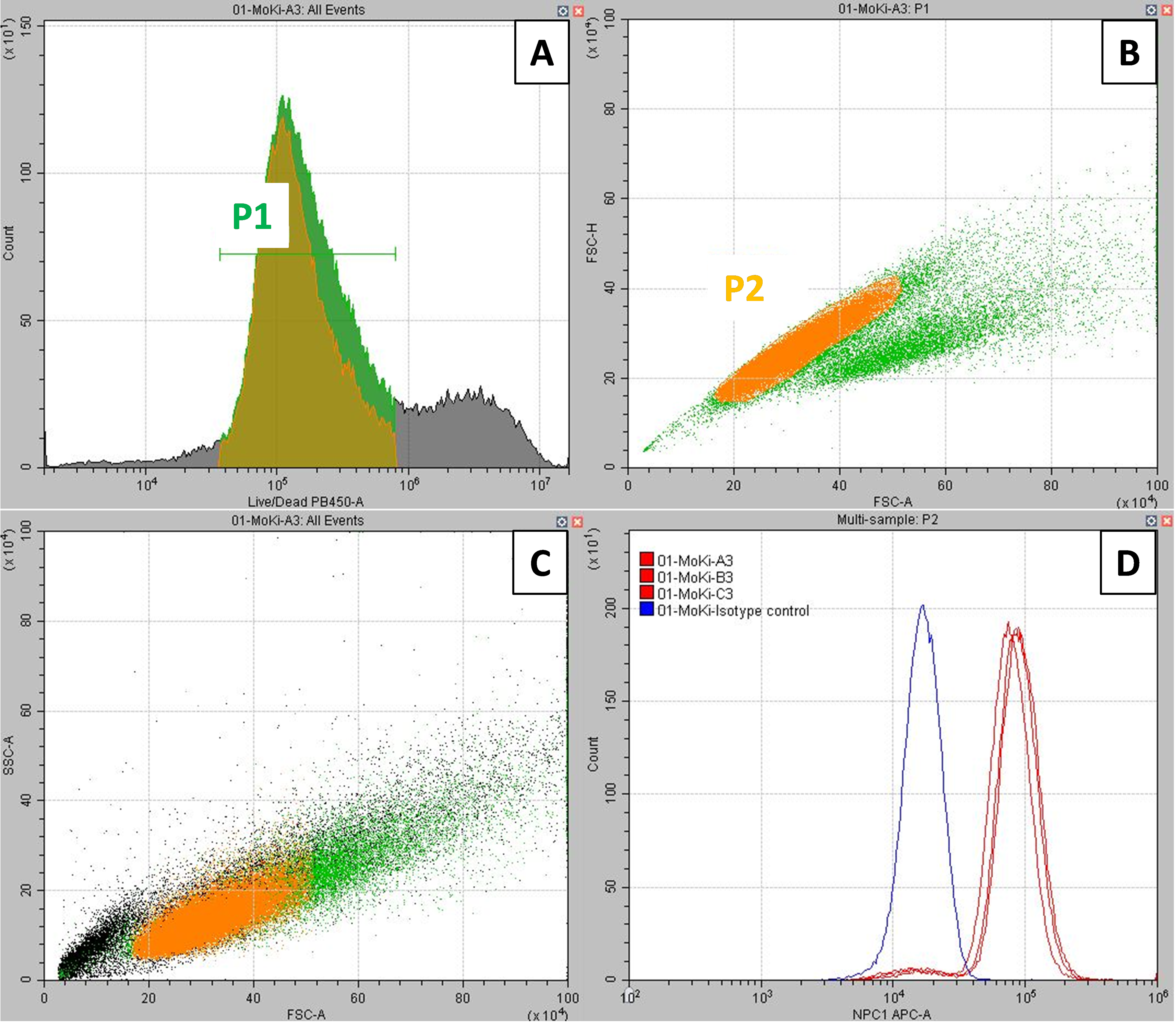

Supplement: S3 Fig — A: Living cells (P1, green); B: singlets (P2, orange); C: Living singlets (orange) in total MoKi cell population; D: relative fluorescence after NPC1 staining (MoKi-A3,B3,C3; red), isotype control (blue). (TIF) [file pntd.0007952.s003.tif]

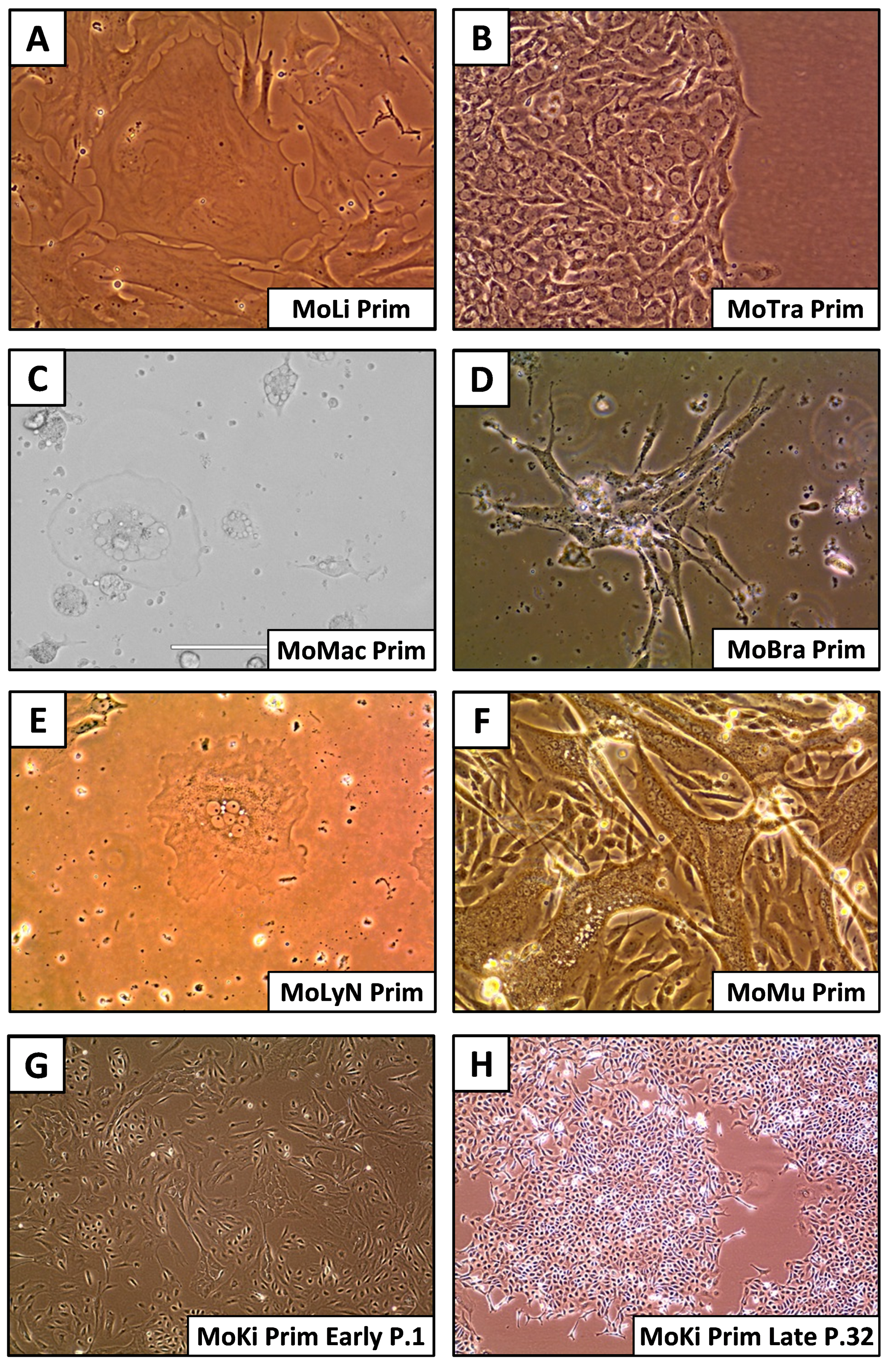

Supplement: S4 Fig — A: Heterogenous liver cell culture with a broad variety of cell types (MoLi Prim); B: Homogenous trachea cell culture with only one recognizable cell type (MoTra Prim); Noticeable cell types–C: Bone marrow-derived macrophages (MoMac Prim); D: Brain cells (MoBra Prim); E: Polymorphonuclear cells (MoLyN Prim); F: Polynuclear syncythia (MoMu Prim); Primary kidney cells with different passage numbers–G: passage one (MoKi Prim Early); H: passage 32, decrease in cell variety (MoKi Prim Late); C: 20x, all other: 40x. (TIF) [file pntd.0007952.s004.tif]

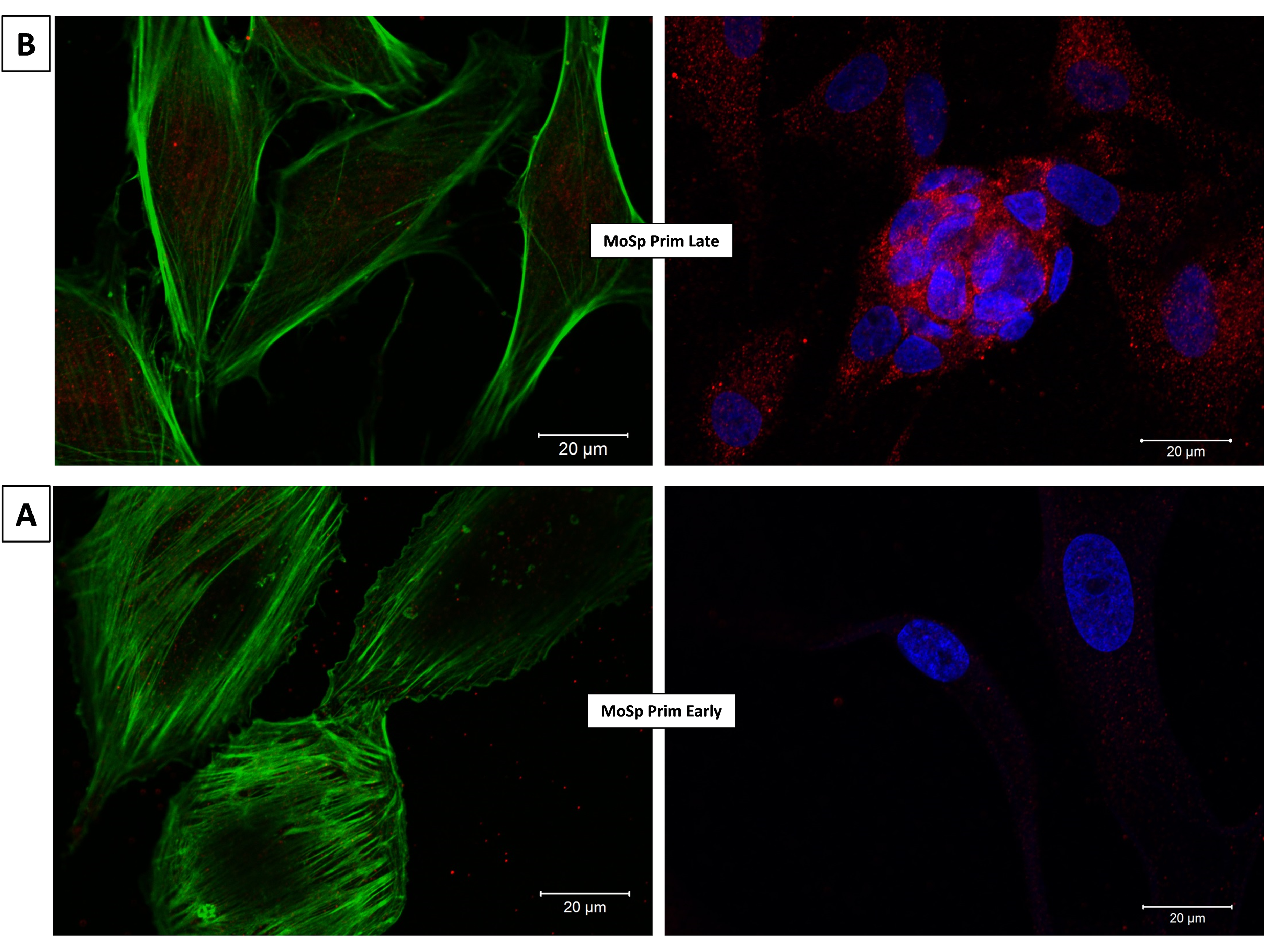

Supplement: S5 Fig — Left column: stained actin filaments (green); Right column: stained NPC1 (red), nucleus stained with DAPI (blue). A: Primary spleen cells in passage 5 (MoSp Prim Early) with low NPC1 expression; B: Primary spleen cells in passage 29 (MoSp Prim Late) with high NPC1 expression. A+B: 63x. (TIF) [file pntd.0007952.s005.tif]

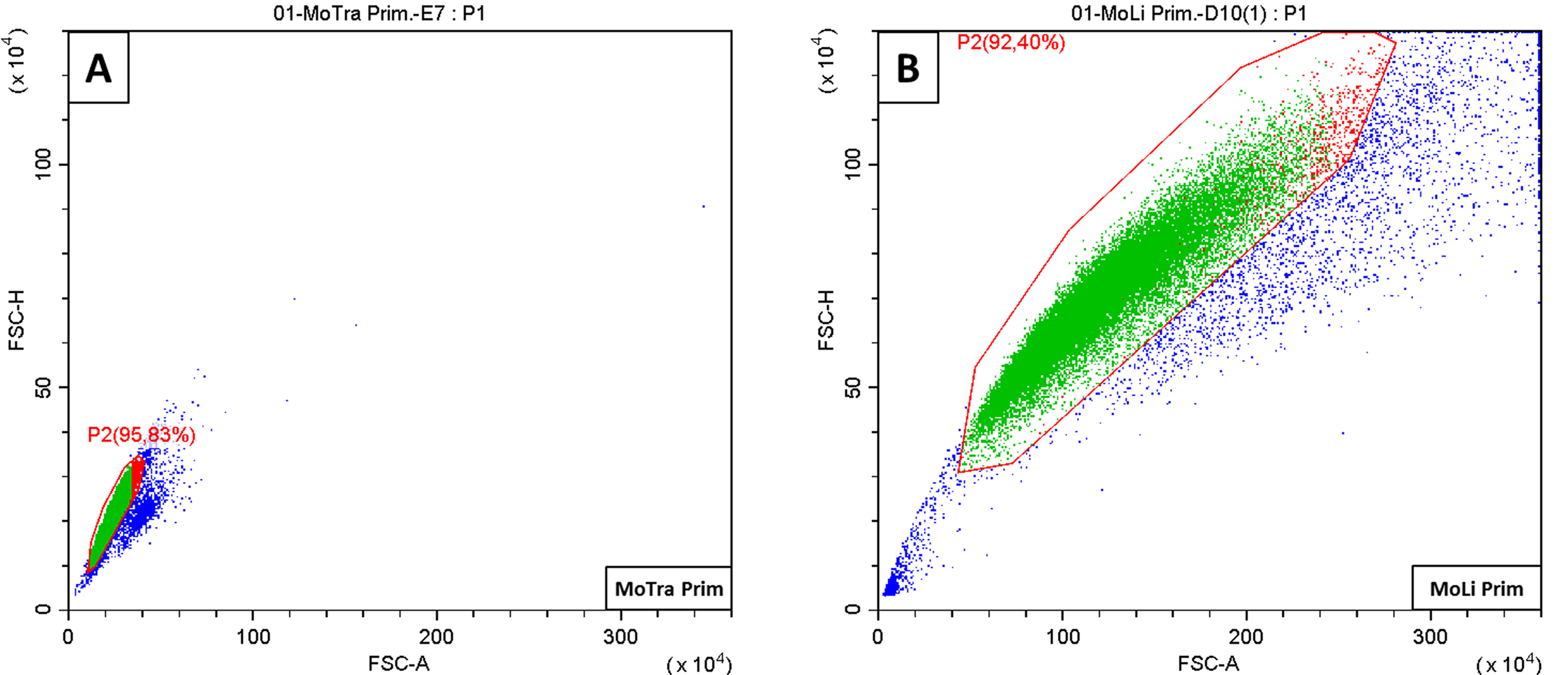

Supplement: S6 Fig — A: homogenous cell culture (MoTra Prim); B: heterogenous cell culture (MoLi Prim), large variety of cell types. (TIF) [file pntd.0007952.s006.tif]

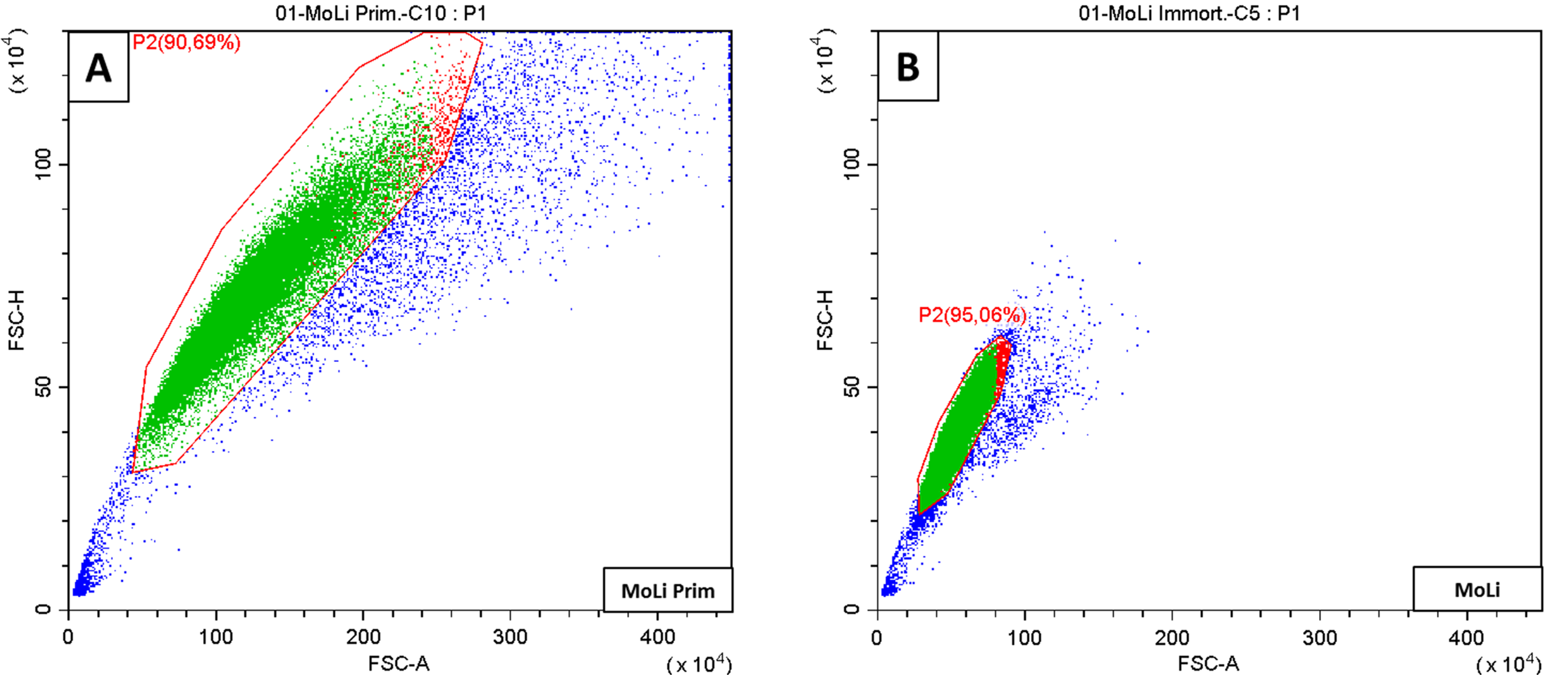

Supplement: S7 Fig — A: large variety of cell types in primary liver cells (MoLi Prim.); B: SV40T immortalized liver cells (MoLi). Immortalization and continuous passaging resulted in relative homogenous cell populations compared to primary liver cell cultures. (TIF) [file pntd.0007952.s007.tif]

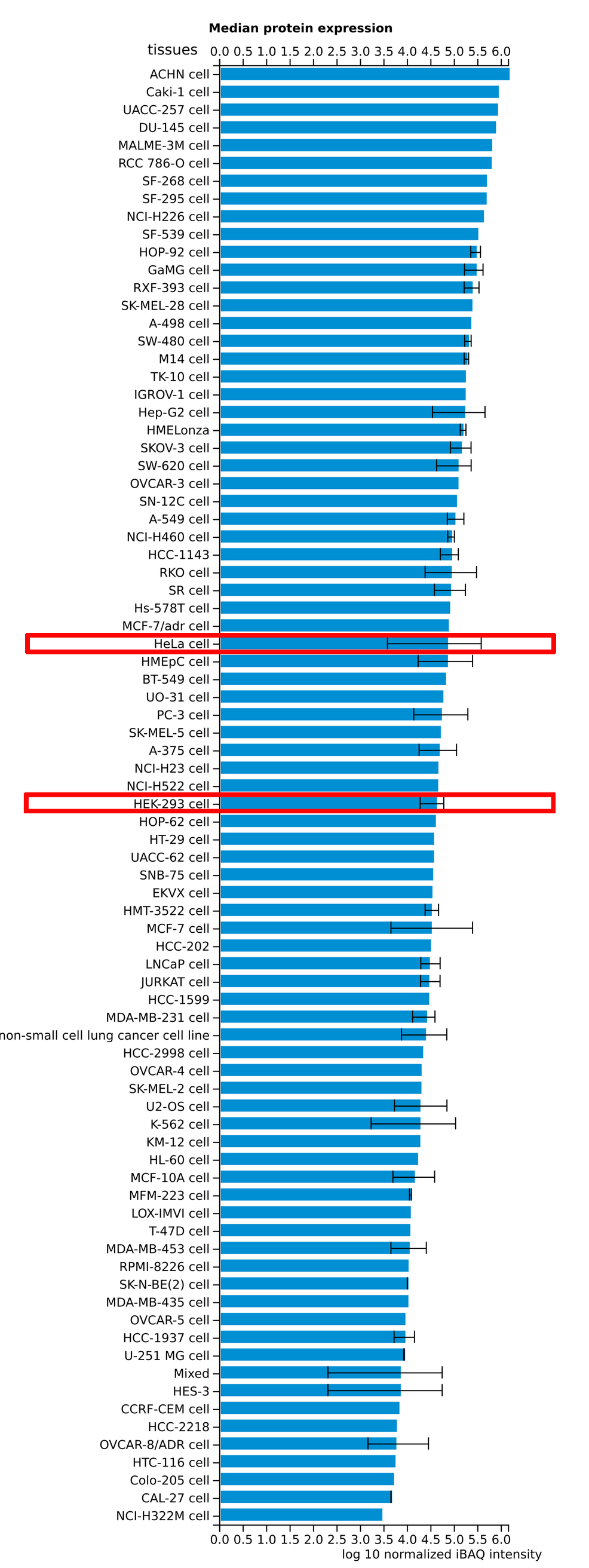

Supplement: S8 Fig — The amount of expressed NPC1 in HeLa and HEK293 cells (red box) is lower than in several other human cell lines. Protein expression is shown as log10 normalized iBAQ intensity; data available on Proteomics DB [37]. (TIF) [file pntd.0007952.s008.tif]
