# Supplementary material for: Utility of primary cells to examine NPC1 receptor expression in Mops condylurus, a potential Ebola virus reservoir
Source: PLoS Negl Trop Dis. 2020 Jan 21;14(1):e0007952. doi: 10.1371/journal.pntd.0007952 (PMC6994141; doi:10.1371/journal.pntd.0007952)
Supplement: S1 Supporting Information — (DOCX) [file pntd.0007952.s009.docx]

**Supporting Information:**

**Utility of primary cells to examine NPC1 receptor expression in Mops condylurus, a potential Ebola virus reservoir**

**S1 Supporting Information. Establishment of Bat Primary Cell Cultures**

MoKi Prim, NyKi Prim, MoTes Prim*, MoLyN Prim*, MoBra Prim* and MoSp Prim*

5 ml Dulbeccos’s Modified Eagle Medium / Nutrient Mixture F12 (DMEM/F12) (D8437, Sigma-Aldrich) at 37°C containing 2 x Antibiotic-Antimycotic and 15%* FBS was pipetted into a sterile petri dish. A kidney, testicle, lymph node, brain or spleen from *M. condylurus* or a kidney from *N. noctula* was squeezed through a cell strainer (EASYstrainer, 100 µm, Greiner) into the medium using a sterile plunger of a 2 ml syringe (Braun). Cells were washed with 15 ml medium, pelleted at 800 g for 5 min, resuspended in 2 ml medium and transferred to a six-well plate (Nunc).

* For culturing of MoTes Prim, MoLyN Prim, MoBra Prim and MoSp Prim cells all cell culture multidishes and flasks were collagen I-coated (BioCoat, Corning). For cultivation of MoTes Prim, MoLyN Prim and MoBra Prim cells the FBS concentration in the growth medium was 20% in the beginning and was reduced to 15% after the first passage.

MoSk Prim

The flying membrane of a *M. condylurus* bat was washed intensively with PBS containing 1 x Antibiotic-Antimycotic. A scalpel was then used to scrape the membrane and collect skin cells, which were transferred directly into a collagen I-coated six-well plate. We used DMEM/F12 medium containing 2 x Antibiotic-Antimycotic and 20% FBS in the beginning. FBS concentration was reduced to 15% after the first passage.

MoMu Prim

The pectoral muscle of a *M. condylurus* bat was dissected on a petri dish into pieces smaller than 1 mm^3^. The pieces of muscle tissue were transferred to a 15 ml conical tube with 2 ml digest medium, consisting of DMEM (41965, Thermo Fisher Scientific) with 0.3 U/ml Collagenase A (10103578001, Sigma-Aldrich) and 2.4 U/ml Dispase I (P3417, Sigma-Aldrich). The tissue pieces were incubated for 30 min at 37°C. Afterwards, 10 ml cell culture medium (DMEM/F12 medium containing 2 x Antibiotic-Antimycotic and 20% FBS) was added followed by filtration through a 100 µm cell strainer. Cells were pelleted at 350 g for 5 min, resuspended in cell culture medium and transferred to a collagen I-coated six-well plate. The FBS concentration was reduced to 15% after the first passage.

MoHe Prim and MoLu Prim

The heart and lung respectively of a *M. condylurus* bat was dissected on a petri dish into pieces smaller than 5 mm^3^. The tissue pieces were transferred to a 15 ml conical tube with 2 ml digest medium, consisting of DMEM/F12 containing 0.3 U/ml Collagenase A. After 30 min at 37°C the cell solution was poured into a 100 µm cell strainer and the remaining tissue pieces were squeezed through the mesh using a sterile plunger of a 2 ml syringe. Cells were then pelleted at 800 g for 5 min, resuspended in DMEM/F12 containing 2 x Antibiotic-Antimycotic and 15% FBS and finally transferred into a six-well plate.

MoTra Prim

The trachea of a *M. condylurus* bat was cleaned and all other attached tissues were removed thoroughly. Afterwards the organ was chopped into thin rings of about 1 mm. Each trachea ring was transferred into 50 µl DMEM/F12 containing 2 x Antibiotic-Antimycotic and 15% FBS in one well of a 24-well plate (Nunc). A low volume of medium prevented the trachea rings from floating and reduced movement of the tissue explant in the initial phase of culture. After 72 hours, 50 µl medium per well was added when the outgrowth of primary trachea cells could be observed and another 200 µl medium per well was added on day 4, 5 and 6. The cells were then inspected daily and the medium was changed on every second day. Trachea rings were removed on day 8. When cells reached 90% confluency they were expanded.

MoLi Prim

The liver of a *M. condylurus* bat was washed intensively in a petri dish with 40 ml Hepatocyte Wash Medium (17704024, Gibco) containing 1 x Antibiotic-Antimycotic. Then the liver was perfused for 10 min with 5 x 3 ml Liver Digest Medium (17703034, Gibco) using a 18G hypodermic needle (Braun). The perfused liver was transferred into a new petri dish with 10 ml William’s E Medium, GlutaMAX Supplement (32551020, Gibco) containing 2 x Antibiotic-Antimycotic and 15% FBS at 4°C [Liver Cell Culture Medium, LCCM]. Using a cell scraper (99002, TPP) gentle pressure on the perfused liver lead to release of liver cells into the medium. The solution was filtered through a cell strainer (100 µm) and diluted with 25 ml ice-cold LCCM. Cells were pelleted at 400 g for 3 min at 4°C and washed four times with 25 ml ice-cold LCCM. Finally cells were resuspended in 10 ml LCCM at 37°C and transferred into a six-well plate (Nunc).

MoMac Prim

We generated bone marrow-derived macrophages from *M. condylurus* using a modified protocol described previously [1].

Production of *P. alecto* CSF-I:

NyKi Prim cells were transfected for 15 min with 5 µg CSF-I plasmid DNA using Lipofectamine 3000 Transfection Kit (L3000001, Invitrogen) with a ratio (Lipofectamin:DNA) of 5:1. Transfection was confirmed with a reporter GFP. Finally the supernatant was collected after 72 hours and sterile filtered before usage.

Macrophage Cell Culture:

*M. condylurus* bone marrow cells in 1 ml Recovery Cell Culture Freezing Medium were diluted slowly with 1 ml Iscove’s Modified Dulbecco’s Medium (IMDM) (L0190-500, Biowest) containing 10% FBS, 1 x Antibiotic-Antimycotic and 40 µM β-Mercaptoethanol (4227.3, Carl Roth) (Macrophage Medium). Cells were then transferred into a 15 ml conical tube with 10 ml Macrophage Medium and pelleted at 400 g for 5 min. Subsequently the cells were resuspended in 3 ml Macrophage Growth Medium consisting of Macrophage Medium with 10% P. alecto CSF I-containing supernatant and 5 x Macrophage Growth Supplement (MaGS) (1972, ScienCell). Finally the cell suspension was transferred into a non-treated polystyrene petri dish (430588, Corning) and then incubated for five days avoiding any movements. At day five 2 ml Macrophage Growth Medium was added and at day nine 3 ml was exchanged. At day 13 MoMac Prim cells were ready for immunofluorescence staining and confocal microscopy.
